# The complete chloroplast genome of *Epimedium flavum* Stearn (Berberidaceae)

**DOI:** 10.1080/23802359.2021.1920500

**Published:** 2021-07-27

**Authors:** Hui Luo, Qiong Liang, Ruoqi Huang, Jingzhou Dong, Yanjun Zhang

**Affiliations:** a School of Modern Industry for Selenium Science and Engineering, Wuhan Polytechnic University, Wuhan, People’s Republic of China; b Key Laboratory of Plant Germplasm Enhancement and Specialty Agriculture, Wuhan Botanical Garden, Chinese Academy of Sciences, Wuhan, People’s Republic of China

**Keywords:** *Epimedium flavum* Stearn, chloroplast genome, Berberidaceae

## Abstract

*Epimedium flavum* Stearn, which belongs to Berberidaceae, is mainly distributed in the Sichuan province of China. In this study, the complete chloroplast genome of *E. flavum* was reported for the first time. The whole genome of *E. flavum* was 159,134 bp in length, and revealed a typical quadripartite structure, including two copies of an inverted repeat (IR) region of 27,735 bp separating a large single-copy region (LSC, 86,576 bp) and a small single-copy region (SSC, 17, 088 bp). The chloroplast genome contained 112 unique genes, including 78 protein-coding genes, 30 tRNA genes, and 4 rRNA genes. Phylogenetic analysis showed that *E. flavum* of series *Davidianae* was firstly clustered with *E. brevicornu* of ser. *Brachyerae*.

*Epimedium* L. contains about 62 species, which are discontinuously distributed in the Mediterranean region and eastern Asia (Ying et al. 2011; Zhang et al. 2020). As the diversity center of *Epimedium*, China possesses about 52 species of the genus and has used *Epimedium* plants as traditional medicine ‘Herba Epimedii’ for more than 2000 years (Zhao et al. 2012). Modern pharmacological studies show that Herba Epimedii can be widely used against sexual dysfunction, osteoporosis, and inflammation (Ma et al. 2020). However, *Epimedium* has abundant variations in morphology and medicinal ingredients, which greatly increases the difficulty of species identification (Zhang et al. 2016). In recent years, with the development of modern molecular techniques, the chloroplast genome has been widely used to study the genetic diversity and phylogenetic relationship in plant species (Yang et al. 2019). In this study, we sequenced the complete chloroplast genome of *E. flavum*, aiming to provide more valuable information about the phylogenetic relationships of *Epimedium* species.

In this study, the *E. flavum* samples were collected from the Tianquan County, Sichuan, China (102°16′E, 30°06′N), and a *Yanjun Zhang 566* (HIB) voucher was deposited at the Herbarium of Wuhan Botanical Garden, Chinese Academy of Sciences (HIB). The genomic DNA was extracted from the fresh leaves using the modified CTAB method (Doyle and Doyle 1987). Genome sequencing was sequenced using Illumina Novaseq PE150, and 150 bp paired-end reads were generated. The filtered reads were assembled using the program GetOrganelle v1.7.2a with *E. acuminatum* chloroplast genome (GenBank accession number: NC_029941) as a reference (Jin et al. 2020). The chloroplast genome sequence was annotated through the online program CPGAVAS 2, followed by manual correction (Shi et al. 2019). The chloroplast genome sequence of *Epimedium flavum* was submitted to the NCBI database with an accession number (MW467895).

The genome sequence of *E. flavum* was 159,134 bp in length, and displayed a typical quadripartite structure, including two copies of an inverted repeat (IR) region of 27,735 bp separating a large single-copy region (LSC, 86,576 bp) and a small single-copy region (SSC, 17,088 bp). The overall GC content of chloroplast genomes was 38.81%, while the GC content in LSC IR and SSC regions was 37.29%, 43.03%, and 32.79%, respectively. The chloroplast genome of *E. flavum* contained 112 unique genes, including 78 protein-coding genes, 30 tRNA genes, and 4 rRNA genes. Among these genes, six tRNA genes and nine protein-coding genes contained one intron, while three genes contained a pair of introns.

To identify the phylogenetic relationship of *E. flavum*, 15 complete chloroplast genomes of Berberidaceae species were downloaded from the NCBI GenBank database. The sequences were aligned using MAFFT v7 (Katoh et al. 2017), and the maximum-likelihood tree was constructed with 1000 bootstrap replicates using raxmlGUI1.5b (v8.2.10) (Silvestro and Michalak 2012; Katoh et al. 2017) (Figure 1). Phylogenetic analysis showed that *E. koreanmum* of sect. *Macroceras* was separated from all the twelve species of the sect. *Diphyllum* of *Epimedium*. *E. flavum* of series *Davidianae* was firstly clustered with *E. brevicornu* of ser. *Brachyerae*, both of which are distributed in the Sichuan Basin, next to the eastern Tibet Plateau. The complete chloroplast genome of *E. flavum* contributes to the phylogenetic and evolutionary analysis of *Epimedium* species.

**Figure 1.**
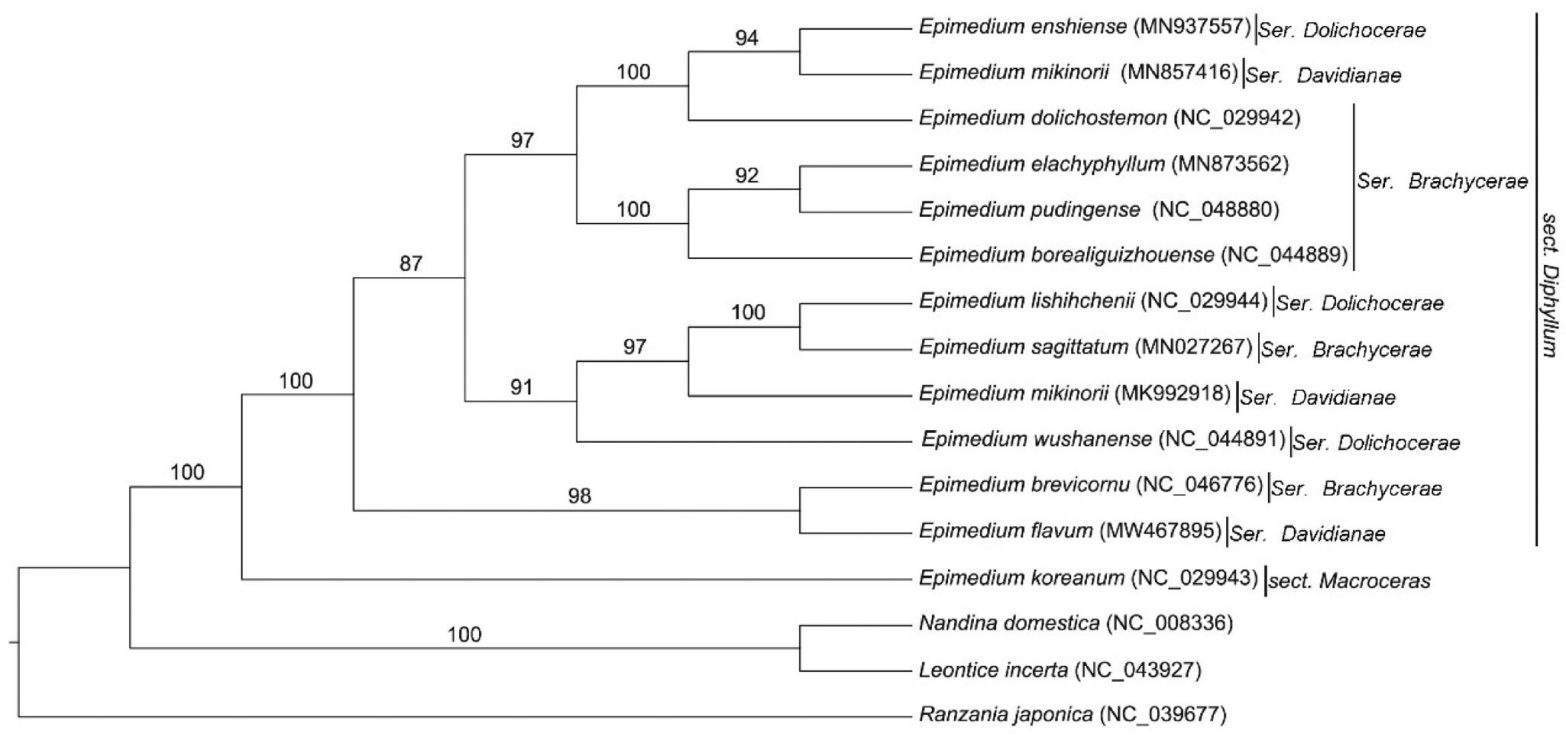
The maximum likelihood (ML) phylogenetic tree based on the complete chloroplast genome of 16 species, with *Ranzania japonica* as an outgroup. Numbers above the lines represent ML bootstrap values.

## Data Availability

The genome sequence data that support the findings of this study are openly available in GenBank of NCBI at (https://www.ncbi.nlm.nih.gov/) under the accession no. MW467895. The associated BioProject, SRA, and Bio-Sample numbers are PRJNA714816, SRR13983522, and SAMN18318609, respectively.
